# Investigation of the Anisotropy of 4H-SiC Materials in Nanoindentation and Scratch Experiments

**DOI:** 10.3390/ma15072496

**Published:** 2022-03-28

**Authors:** Suhua Shi, Yiqing Yu, Ningchang Wang, Yong Zhang, Weibin Shi, Xinjiang Liao, Nian Duan

**Affiliations:** 1 College of Mechanical Engineering and Automation, National Huaqiao University, Xiamen 361021, China; suhuashi@foxmail.com (S.S.); yyqing@hqu.edu.cn (Y.Y.); zhangyong@hqu.edu.cn (Y.Z.); 15484@hqu.edu.cn (W.S.); xinjiangliao@sina.cn (X.L.); 2 Zhengzhou Research Institute for Abrasives & Grinding Co., Ltd., Zhengzhou 450001, China; wangningchang@126.com; 3 State Key Laboratory of Super-Abrasives, Zhengzhou 450001, China

**Keywords:** 4H-SiC, anisotropy, nanoindentation and scratch tests, scratches, morphologies

## Abstract

Silicon carbide is an ideal material for advanced electronics, military, and aerospace applications due to its superior physical and chemical properties. In order to understand the effect of crystal anisotropy of 4H-SiC on its processability, nanoindentation and nanoscratch tests on various crystallographic planes and orientations were performed and the results outlined in this paper. The results show that the C-plane of 4H-SiC is more rigid, while the Si-plane is more elastic and ductile. Better surface quality may be obtained on the Si-plane in nanoscale abrasive machining. The maximum lateral force, maximum residual depth of the scratch, and maximum crack width on the C- and Si-planes of 4H-SiC are significantly periodic in crystallographic orientations at 30° intervals. The scratch along the <11$\bar{2}$0> direction is more prone to crack expansion, and better machined surface quality is easy to obtain along the <10$\bar{1}0$> directions of C- and Si-planes.

## 1. Introduction

Monocrystalline silicon carbide (SiC) has a series of excellent physical and chemical properties, including high elastic modulus, moderate density, relatively low coefficient of thermal expansion, relatively high thermal conductivity and thermal shock resistance, high specific stiffness, and dimensional and thermal stability, which offer irreplaceable advantages to SiC materials in nanotechnology, bulletproof armor, 4H-SiC based Schottky diodes [1,2], and space telescopes, as well as the extreme environments of aerospace [3,4,5]. The hardness, high brittleness, and low surface energy of SiC materials render them a removal mechanism more of brittle fracture than of plastic deformation. The low machinability creates a bottleneck in their use in high-precision applications [6,7].

**Figure 1:**
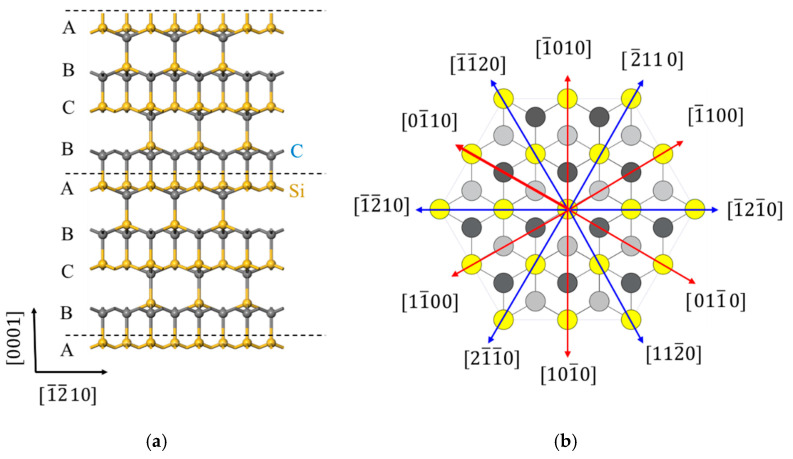
Atomic arrangement of (**a**) (1$\bar{2}$10) and (**b**) (0001) planes in 4H-SiC.

The basic unit in SiC is a tetrahedron of four carbon atoms with a silicon atom in the center, linked through covalent bonding [8]. The most commonly used polytype of SiC is 4H-SiC with a hexagonal lattice structure [9]. The discrepancy in lattice creates anisotropy, which further affects the properties of workpiece materials in terms of plastic mechanics and fracture mechanics. Therefore, the anisotropy of monocrystalline silicon carbide materials causes great variance in the physical and mechanical properties of different crystallographic planes and orientations. Figure 1 shows the atomic arrangements of the (1$\bar{2}$10 and (0001) planes of 4H-SiC. As shown in Figure 1a, on the (1$\bar{2}$10) plane, the C atoms and Si atoms in the [0001] direction of 4H-SiC are cyclically arranged in an … ABCBA … way. As shown in Figure 1b, 4H-SiC has a total of 12 crystallographic orientations on the (0001) crystal plane.

Nanoindentation and scratch technologies are widely used to study the micromechanical properties and deformation and damage mechanisms of materials at the microscale and nanoscale. Some research has been carried out on the anisotropy of monocrystalline silicon carbide. Miyoshi and H. Buckley [10] studied silicon carbide surfaces in sliding contact with diamond, and found that the <10$\bar{1}$0> direction on the basal plane (0001) presented the lowest coefficient of friction and the greatest resistance to abrasion. During sliding, the anisotropic fracture on the basal plane was the surface cracking along (1010), and the fracture on the (1010) and (11$\bar{1}$0) surfaces resulted from the surface cracking along (0001) or (1120) or (1010) and from the subsurface cracking along (1010). Scratch tests by Wang et al. [11] showed that the scratch along the [1$\bar{1}0$0] direction of 6H-SiC single crystals generated the maximum average coefficient of friction while the scratch along the [1̅21̅0] direction the minimum. National Aeronautics and Space Administration (NASA) [12] conducted a validation experiment and obtained in the Young’s modulus a maximum value of 557GPa in the (111) plane of silicon carbide single crystals and a minimum value of 314Gpa in the (100) plane. Through indentation experiments, Cody Kunka et al. [13] concluded that the fracture toughness for <11$\bar{2}$0> direction is 30% lower than that of <10$\bar{1}$0> direction on the basal plane of 4H-SiC. K. Eswar Prasad et al. [14] used nanoindentation to study the anisotropy of 4H-SiC hardness and observed a higher hardness on the (0001) plane when compared with the (1010) plane. The relationship between severing surface edges and crystallographic orientations in the micro-crack-induced severing of 4H-SiC has also been studied. Severing along the <1$\bar{2}\bar{1}$0> direction reduces the severing force by 51.9–64.5% and severing time by 20–33% [15]. The above analysis indicates that most researchers have used indentation and scratch methods to study certain anisotropy of monocrystalline SiC, such as friction coefficient, hardness, elastic modulus, and stress, but they mainly focus on a single index. Little research has been conducted to systematically study the correlation between anisotropic properties of monocrystalline SiC regarding the hardness, elastic modulus, and scratch morphology.

In order to understand the influence mechanism of anisotropy of 4H-SiC during the nanoindentation process, this paper used nanoindentation and nanoscratch tests to investigate the material mechanical properties of different crystallographic planes of monocrystalline 4H-SiC materials as well as the related parameters of different crystallographic orientations such as scratching force and morphology. In addition, the regularity was observed and analyzed, and the correlation was qualitatively elucidated. The divergence between different crystallographic planes and orientation of 4H-SiC was then identified and the reasons explained.

## 2. Experiments

### 2.1. Experimental Platform

Nanoindentation and scratch tests were performed on 4H-SiC using Nano Indenter G200 (Keysight Technologies, Santa Rosa, CA, USA). The parameters of the instrument are shown in Table 1. The maximum loads in XP mode and DCM mode are 500 Mn and 30 Mn respectively.

### 2.2. Experimental Details

**Figure 2:**
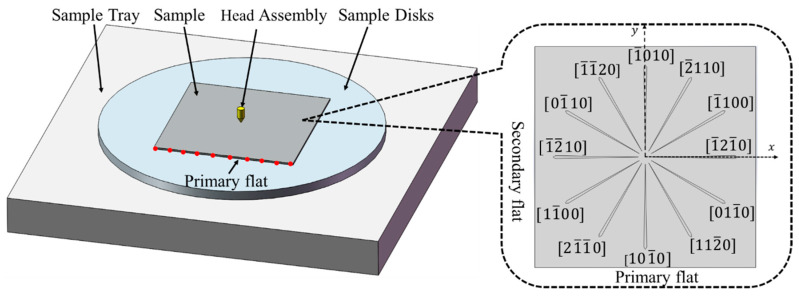
Schematic diagram of 4H-SiC sample preparation and indentation and scratches.

A 2-inch 4H-SiC wafer produced by TankeBlue in Beijing, China, polished on both sides with a thickness of 330 μm and surface roughness $R_{a}<1\;\mathrm{nm},$ was used in the experiments. The direction of the purchased wafers is on axis: <0001> ± 0.5°. Since the sample disks of the nanoindenter allow for a maximum sample diameter of 32 mm, the 2-inch wafer was cut into a 15 mm × 15 mm square sample with a dicing machine. Figure 2 shows the schematic diagram of the 4H-SiC sample. 

The nanoindentation tests were performed on the C- and Si-planes of the 4H-SiC sample using a diamond Berkovich indenter with a fillet radius of 20 nm. The maximum penetration depths were set to 100 nm, 200 nm, 500 nm, 800 nm, 1000 nm, and 1200 nm, and at each depth, tests were repeatedly conducted four times. As is shown in Figure 2, 10 dots were uniformly chosen on the primary flat of the sample under the 10× microscope of the nanoindenter, hence their coordinates in the nanoindenter. Through fitting, a linear equation of the primary flat in the nanoindenter coordinate system was created, and the angle of the [$\bar{1}2\bar{1}0$] direction in the coordinate system was calculated according to the slope of the equation. Therefore, accurate positioning was achieved.

Since the head assembly could only move in the Z-direction, the translation and rotation of the sample were controlled by that of the sample tray during scratching. A conical indenter with a tip fillet radius of 5 μm was used in nanoscratch tests, and the parameters are shown in Table 2. Ramp loading was used with a linear variation of load from 0–200 Mn. Starting from the [$\bar{1}2\bar{1}0]$ direction, scratching was performed counterclockwise along crystallographic orientations at 30° intervals. A total of 12 scratches were made, as is shown in Figure 2. Scratch tests were repeatedly conducted five times on the C- and Si-planes respectively. Taking the scratch center as the origin, the [$\bar{1}2\bar{1}0$] direction as the x-axis, and [$\bar{1}0$10] as the y-axis, the correspondence between crystallographic orientations and angles in the coordinate system would be [$\bar{1}$2$\bar{1}$0] (0°), [$\bar{1}10$0] (30°), [$\bar{2}11$0] (60°), [$\bar{1}0$10] (90°), [$\bar{1}\bar{1}$20] (120°), [$0\bar{1}$10] (150°), [$1\bar{2}$10] (180°), [$1\bar{1}0$0] (210°), [2$\bar{1}\bar{1}$0] (240°), [10$\bar{1}0$] (270°), [11$\bar{2}$0] (300°), and [01$\bar{1}$0] (330°). Crystallographic orientations can be indicated by corresponding angles.

**Figure 3:**
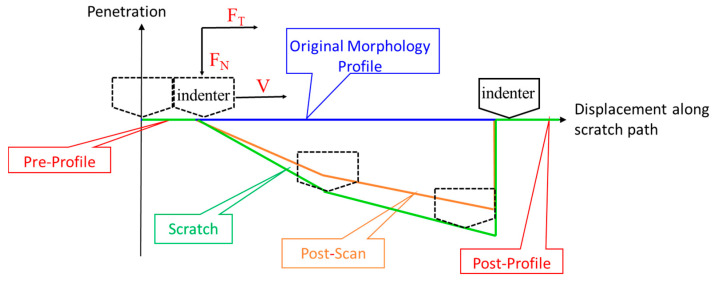
Schematic diagram of the scratch process.

During scratching, the indenter travelled a longer distance than the actual length of the pre-set scratches, because the indenter would pre-profile by travelling a 20% distance before starting the actual scratching and then withdraw to post-profile and travel another 20% distance. The scratch process consisted of three travels of the indenter, i.e., pre-profile, scratch, and post-profile. Figure 3 is a schematic diagram of the scratch process. In step one, the sample surface was pre-profiled with a load of 20 μN for the flatness and the original roughness; the second step was to scratch with a load ranging from 20 μN to the specified maximum value; and in the third step, the final scratching morphology was scanned with a load of 20 Μn. Scratch images were taken with a scanning electron microscope (SU5000, HITACHI, Tokyo, Japan).

### 2.3. Analysis of the Indentation Test Results of Monocrystalline Silicon Carbide Materials

#### 2.3.1. Young’s Modulus and Hardness of the C- and Si-Planes of 4H-SiC

**Figure 4:**
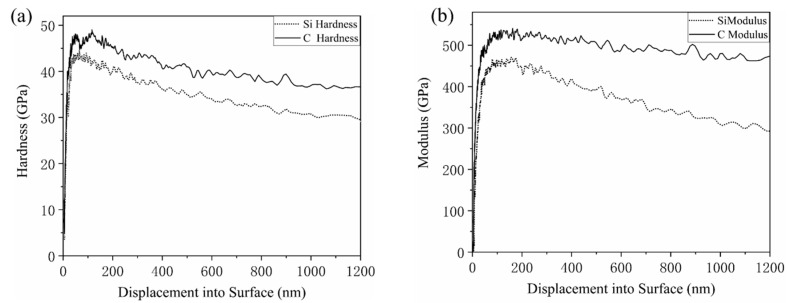
Variation of hardness and elastic modulus of 4H-SiC at 1200 nm. (**a**) Hardness; (**b**) Modulus.

Figure 4 shows the variation of hardness and elastic modulus of 4H-SiC at a penetration depth of 1200 nm. During indentation, the hardness and elastic modulus of both C- and Si-planes decreased with the increase of penetration depth and finally reached a relatively stable stage. This was due to the underestimation of the indentation contact area caused by the abrasion of the indenter tip. The error gradually decreased as the maximum penetration depth increased. The values of hardness and elastic modulus of the C- and Si-planes of 4H-SiC in the 1000–1200 nm section (MN section) when curves of the four tests relatively flattened out were averaged and calculated. For the C-plane, the elastic modulus E = 476.7 Gpa and the hardness H = 37.62 Gpa, and for the Si-plane, the elastic modulus E = 305.5 Gpa and the hardness H = 29.77 Gpa. The elastic modulus of the C-plane was greater than those of the Si-plane.

#### 2.3.2. Load Displacement Curves

**Figure 5:**
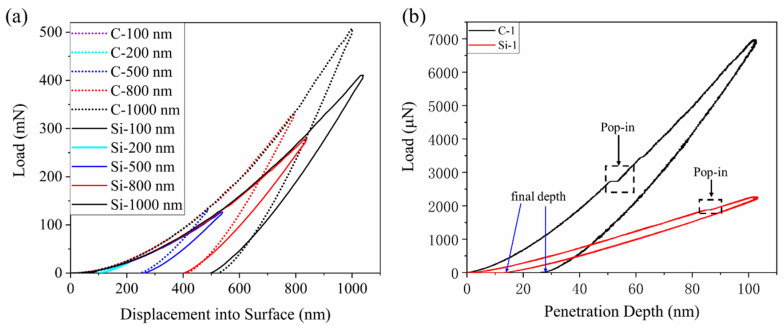
(**a**) Displacement-load curves, (**b**) Displacement-load curves at 100 nm penetration depth.

Figure 5 shows the displacement-load curves of the C- and Si-planes. Figure 5a shows the load-displacement curves of the indentation at the five maximum penetration depths of 100 nm, 200 nm, 500 nm, 800 nm, and 1000 nm. During the loading phase, the curves for both the C- and Si-planes overlapped highly consistently. The load increased with the maximum penetration depth, and when it reached the maximum value unloading started. The unloading curves for different loads were parallel on the C-plane as well as the Si-plane.

Figure 5b shows the displacement-load curves at the maximum penetration depth of 100 nm. The residual depth of indentation was 25.67 nm for the C-plane and 12.22 nm for the Si-plane. At the same depth of penetration, the residual depth for the C-plane was far larger than that of the Si-plane, and the Si-plane claimed more elastic recovery than the C-plane. The load curve of the C-plane is above that of the Si-plane at the same depth during loading, indicating an overall larger load on C-plane than on the Si-plane.

The plastic deformation of 4H-SiC is caused by the directional movement of a large number of atoms following the nucleation and expansion of the dislocation loop [14], which is manifested on the load-displacement curves in the form of pop-in. As is shown in Figure 5, the curve for the C-plane is significantly deflected at 50.77 nm, marking a pop-in in the penetration depth, so h = 50.77 nm. This is the threshold for the elastic-plastic deformation transition of the C-plane. Similarly, pop-in occurs at 84.55 nm on the Si-plane, indicating that the Si-plane is experiencing elastic deformation when h < 84.55 nm, and 4H-SiC is in the plastic deformation stage when h > 84.55 nm. The Si-plane has a higher pop-in value than the C-plane.

### 2.4. Analysis of Nanoscratch Test Results of Monocrystalline Silicon Carbide Materials

#### 2.4.1. Lateral Force

**Figure 6:**
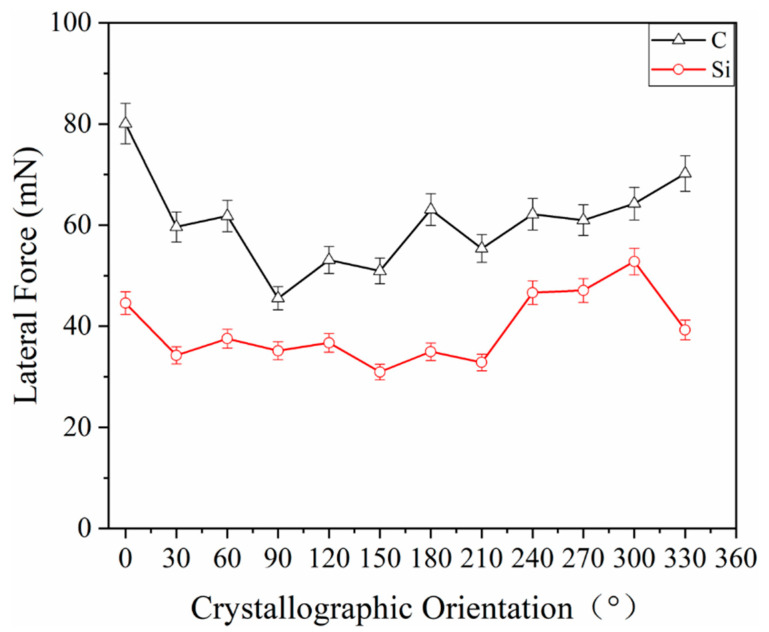
Maximum lateral forces on different crystallographic orientations of 4H-SiC.

Since ramp loading was adopted during the scratch process, the scratch depth and the lateral force would gradually increase as the load increased, until they finally reached the maximum value. Figure 6 shows the curves of maximum lateral forces on different crystallographic orientations, and the overall curve of the C-plane lies above that of the Si-plane, i.e., a larger lateral force was required for scratching on the C-plane than on the Si-plane under the same experimental conditions. Both the curves of the C- and Si-planes fluctuate periodically, where the maximum lateral force is at the peaks of the curve in the <11$\bar{2}$0> direction of the Si-plane, but in the troughs in the <10$\bar{1}0$> direction. The fluctuation of the lateral force of the C-plane is consistent with that of the Si-plane, except for the [$01\bar{1}$0] direction. For the C plane, the lateral force in the [11$\bar{2}$0] direction in the <11$\bar{2}$0> direction is the largest, the lateral force in the [$\bar{1}\bar{1}$20] direction is the smallest, and the lateral force in the [01$\bar{1}$0] direction in the <10$\bar{1}0$> direction is the maximum, and the lateral force in the [$\bar{1}0$10] direction is the minimum. For the Si plane, the lateral force in the [11$\bar{2}$0] direction in the <11$\bar{2}$0> direction is the largest, the lateral force in the [$1\bar{2}$10] direction is the smallest, the lateral force in the [10$\bar{1}0$] direction in the <10$\bar{1}0$> direction of the Si plane is the largest, and the lateral force in the [$0\bar{1}$10] direction is the smallest. The mean lateral force in the <11$\bar{2}$0> direction of the C-plane is 64.074 Mn, which is greater than the mean lateral force in the <10$\bar{1}0$> direction of 57.116 Mn. The mean lateral force in the <11$\bar{2}$0> direction of the Si-plane is 42.206 Mn, which is larger than the mean lateral force in the <10$\bar{1}0$> direction of 36.594 Mn.

Scratch friction coefficient $f_{s}=\frac{F_{T}}{F_{N}}$, where$\;F_{T}$ is the scratch friction force, and $F_{N}$ is the forward load, i.e., $f_{s}$ is proportional to $F_{T}$. Since ramp loading was adopted in the test, the forward load is of linear variation ranging 0–200 Mn. When $F_{N}$ = 200 Mn, $f_{s}=\frac{1}{200}F_{T}$, so the variation of the friction coefficient is consistent with that of the scratch friction force.

#### 2.4.2. Scratch Morphology of 4H-SiC under SEM

**Figure 7:**
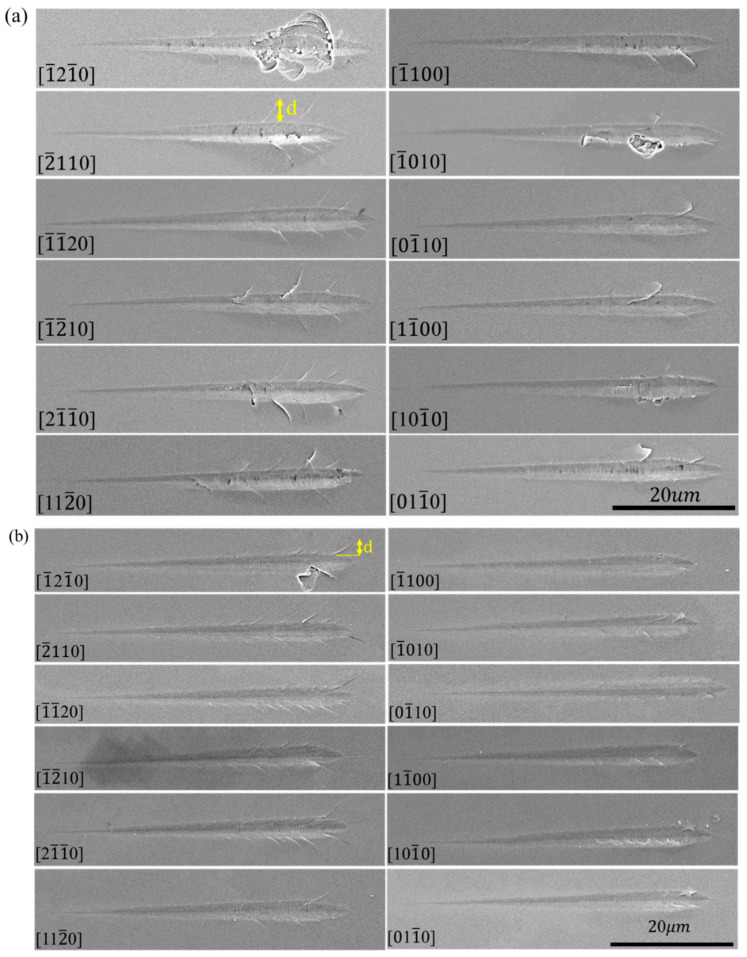
Scratch morphologies in different crystallographic orientations of the C- and Si-planes: (**a**) C-plane, (**b**) Si-plane.

Figure 7 shows the representative residual morphologies of the scratches of 12 crystallographic orientations of the C- and Si-planes of 4H-SiC under SEM. Elastic deformation occurred in the initial stage of scratching, hence the complete recovery of scratch morphologies. Then smooth grooves started to appear. The scratch depth gradually increased with the forward load, and tearing started to appear in the grooves. As the scratch depth went up further, the tearing in the grooves widened, and cracks began to flank the scratches. The deeper the scratches, the longer the cracks. Some cracks deflected, and materials even peeled off in certain directions.

Figure 7a shows the scratch morphologies in different crystallographic orientations on the C-plane. Cracks flanking the scratches in the <11$\bar{2}$0> direction outnumbered those in the <10$\bar{1}0$> direction. The same occurred on the Si-plane as is shown in Figure 7b. The flanking cracks in the [$\bar{1}$2$\bar{1}$0] and [$\bar{1}0$10] directions of the C-plane and the [$\bar{1}$2$\bar{1}$0] direction of the Si-plane stretched and connected, resulting in the spalling of materials. Cracks on the C-plane were deeper than those on the Si-plane, and cracks in the <10$\bar{1}0$> direction of the Si-plane occurred inside the grooves and rarely extended to the outside.

**Figure 8:**
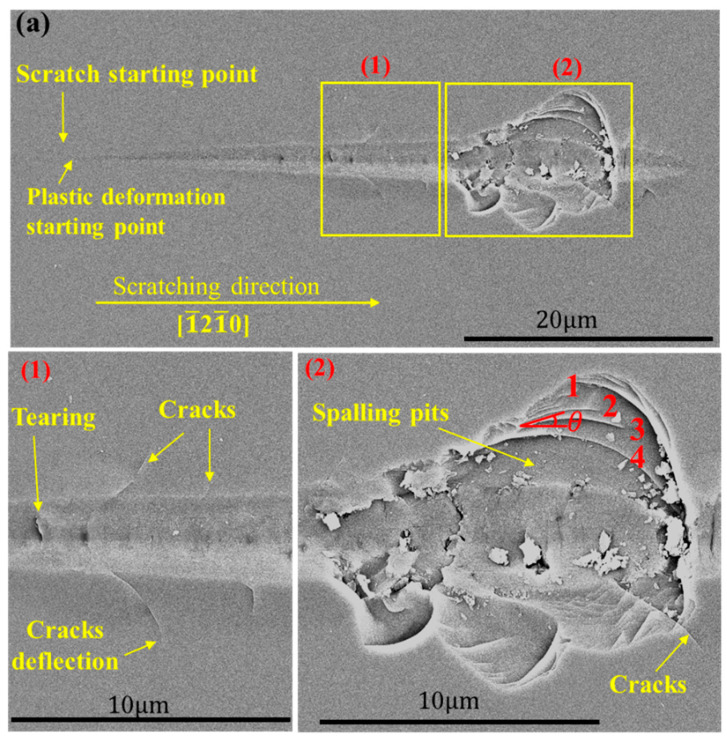

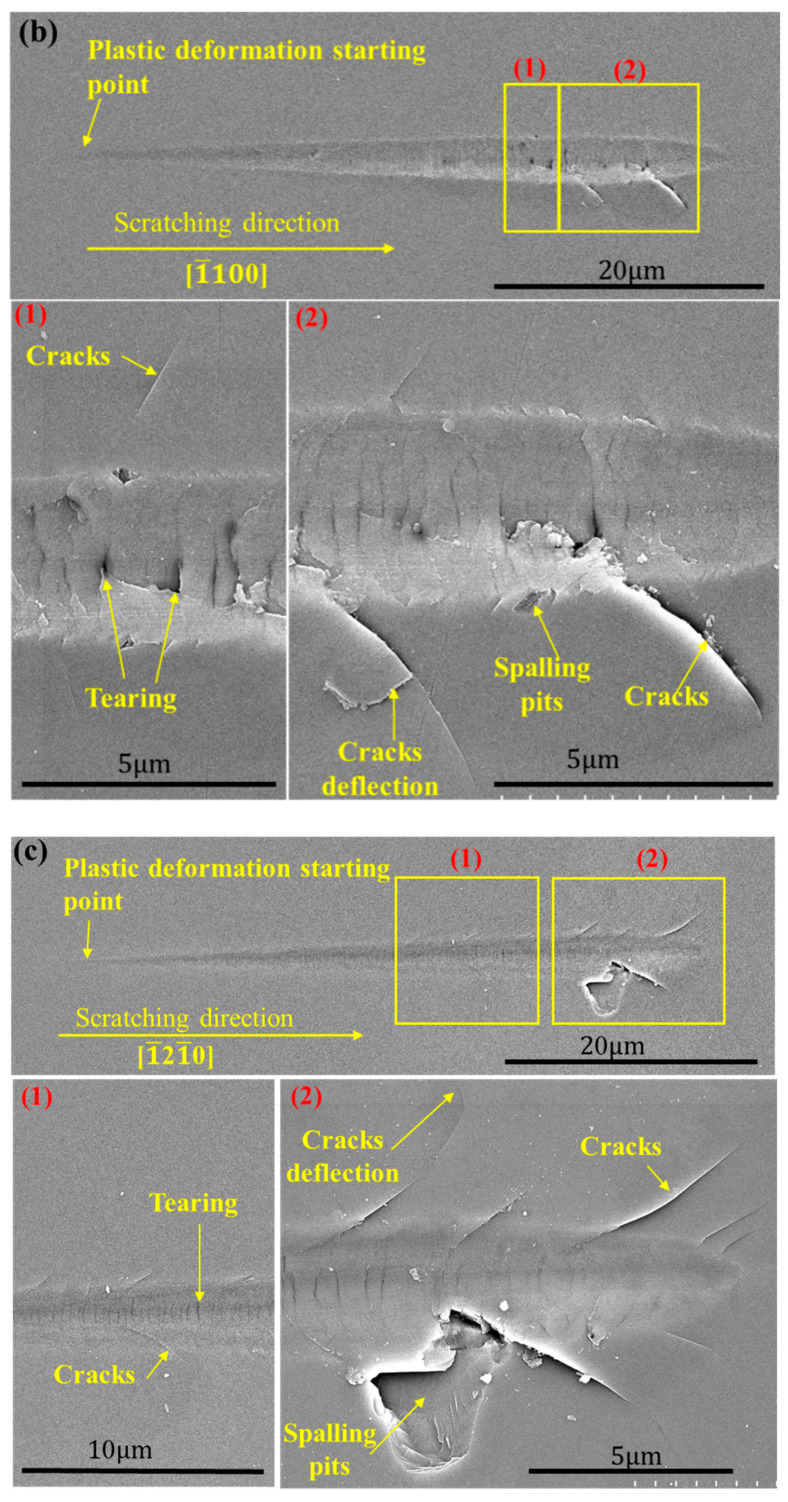

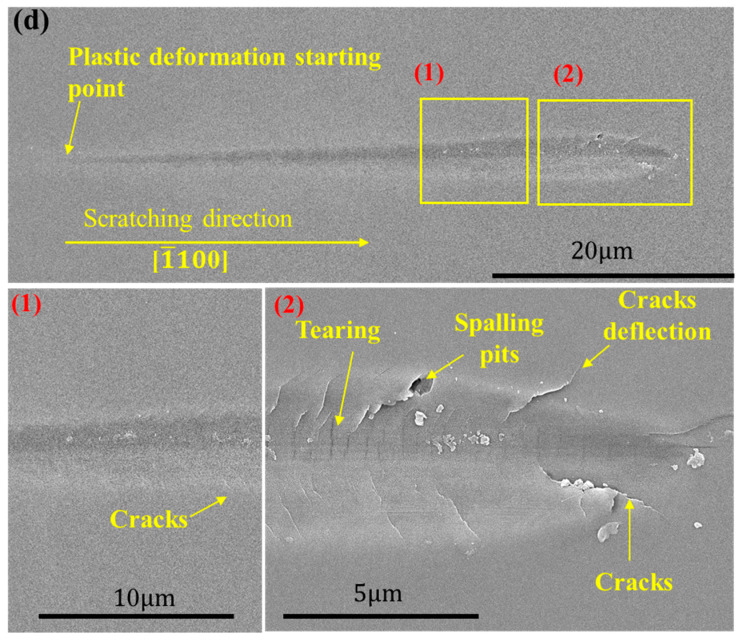
Scratch morphologies in the [$\bar{1}$2$\bar{1}$0] and [$\bar{1}10$0] directions of the C- and Si-planes. (**a**) [$\bar{1}$2$\bar{1}$0] direction of the C-plane; (**b**) [$\bar{1}10$0] direction of the C-plane; (**c**) [$\bar{1}$2$\bar{1}$0] direction of the Si-plane; (**d**) [$\bar{1}10$0] direction of the Si-plane.

The above shows that similar morphologies appeared in the <11$\bar{2}$0> direction and in the <10$\bar{1}0$> direction. In addition, materials in the [$\bar{1}$2$\bar{1}$0] direction peeled off. Therefore, morphologies in the [$\bar{1}$2$\bar{1}$0] and [$\bar{1}10$0] directions are chosen for specific analysis. Figure 8a,b are the detailed views of the [$\bar{1}$2$\bar{1}$0] and [$\bar{1}10$0] directions of the C-plane. Between the starting point of the scratch and the plastic deformation stands the elastic deformation stage, with no grooves in the morphology. As in Figure 8a, massive spalling occurred in the [$\bar{1}$2$\bar{1}$0] direction of the C-plane, in the place of arrayed line bumps. It can be seen from the figure that the fragmented materials were relatively consistent, all in the form of steps. The angles θ between the lines 1, 2, 3, and 4 and the scratch directions were measured to be 19.537°, 19.597°, 19.365°, and 20.037°. No spalling was spotted in the [$\bar{1}10$0] direction of the C-plane. In addition, for the C-plane, more cracks flanked the groove in the [$\bar{1}$2$\bar{1}$0] direction than in the [$\bar{1}10$0] direction, while the tearing was densely packed in the groove of the [$\bar{1}10$0] direction with a greater length when compared with that of the [$\bar{1}$2$\bar{1}$0] direction. Materials also tore more evidently inside the groove of the former direction. With further loading, chunks of materials first peeled off the right side of the groove, and the flanking crack lengthened and deflected.

In Figure 8c, the lower side of the [$\bar{1}$2$\bar{1}$0] direction of the Si-plane peeled off, with an area smaller than that of the C-plane, but no spalling occurred in the [$\bar{1}$2$\bar{1}$0] direction of the Si-plane (Figure 8d). More cracks flanked the groove in the [$\bar{1}$2$\bar{1}$0] direction of the Si-plane than in the [$\bar{1}10$0] direction, while the groove in the [$\bar{1}10$0] direction was densely packed with longer tearing when compared with the [$\bar{1}$2$\bar{1}$0] direction, and with minor spalling of materials.

The morphology of the scratched surface can be roughly divided into three phases:

1. Elastic phase. No mark is left on the scratched surface and the deformation is fully recovered.

2. Plastic phase. The interior of the groove is relatively smooth, and the width and depth of the groove increase with the load.

3. Brittle-ductile transition phase. Tearing first appears in the scratched groove, followed by micro-cracks in the groove and at the edges. As load increases, cracks lengthen in a direction influenced by the crystallographic orientation. Materials inside the groove tear more apart, and cracks on the flanks stretch and deflect. With a further increase in the load, the flanking cracks continue to expand and connect resulting in the spalling of materials.

#### 2.4.3. Deformation Length in the Elastic Phase

**Figure 9:**
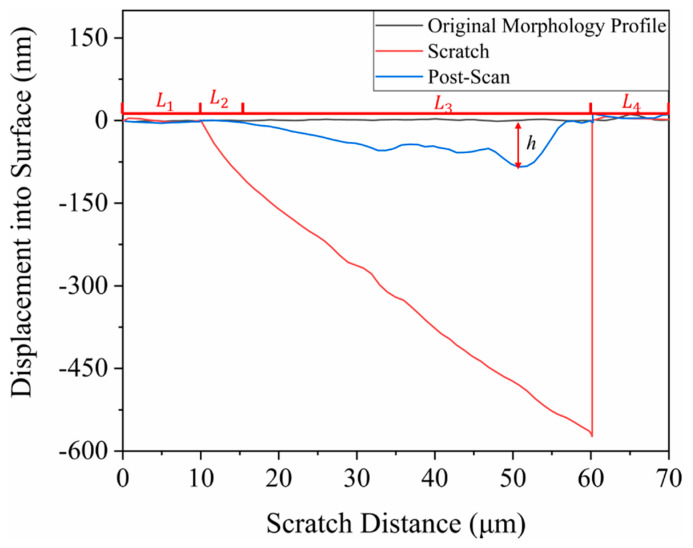
Displacement-depth curves.

Figure 9 shows the displacement-depth curves in the [$\bar{1}10$0] direction of the C-plane, where L1 and L4 are the unloaded regions, and L2 and L3 are the scratch regions. The scratch deepened with the increase of displacement. In the L2 region, the scratch depth increased approximately linearly with the increase of load. In the scan curve, the final curve of the scratch depth coincided with the original morphology, approximately zero in the depth, and therefore can be regarded as elastic deformation. In the L3 region, the post-scan curve claimed a scratch depth lower than the original surface and fluctuated greater with the increase of the scratch depth. This may be caused by the plastic deformation, cracking, chipping, and spalling of the silicon carbide materials due to the increase of load. The deepest point of scratch was 85.455 nm at a scratch distance of 51.75 um. Based on the displacement-depth curves, the length of elastic deformation of the scratch in each crystallographic orientation of the five tests was calculated. The average length of the elastic deformation was 6.149 μm on the C-plane, shorter than 7.854 μm on the Si-plane.

#### 2.4.4. Maximum Scratch Depth

**Figure 10:**
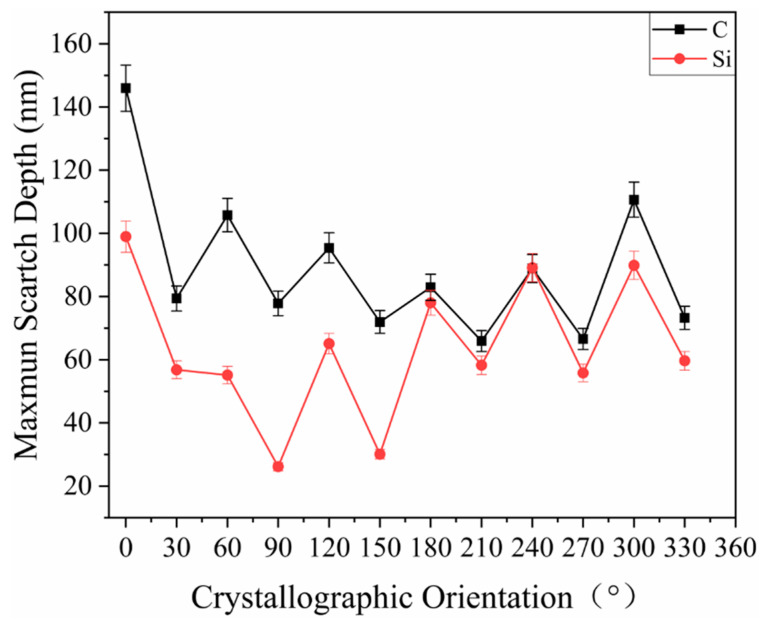
Maximum residual depth of scratches.

Figure 10 shows the curves of the maximum residual depth of the scratch in each crystallographic orientation. As is shown in Figure 10, the curve of the maximum scratch depth of the C-plane lies above that of the Si-plane, i.e., the C-plane had a greater scratch residual depth than the Si-plane, which was consistent with the conclusion of the residual depth of penetration in the indentation tests. This was because the elastic modulus and hardness of the C-plane were greater than those of the Si-plane. The curves of the maximum residual depth of scratches on the C- and Si-planes fluctuate periodically, reaching peaks in the <11$\bar{2}$0> direction, and troughs in the <10$\bar{1}0$> direction. The [11$\bar{2}$0] residual depth of scratches is the largest in the <11$\bar{2}$0> direction on the C and Si planes. The average maximum residual depth of scratches in the <11$\bar{2}$0> direction of the C-plane is 104.930 μm, which is greater than the average maximum residual depth of scratches in the <10$\bar{1}0$>direction of 72.488 μm. The average maximum residual depth of scratches in the <11$\bar{2}$0> direction of the Si-plane is 79.377 μm, which is larger than the average maximum residual depth of scratches in the <10$\bar{1}0$> direction of 47.802 μm.

#### 2.4.5. Maximum Width of Cracks Flanking the Scratch

**Figure 11:**
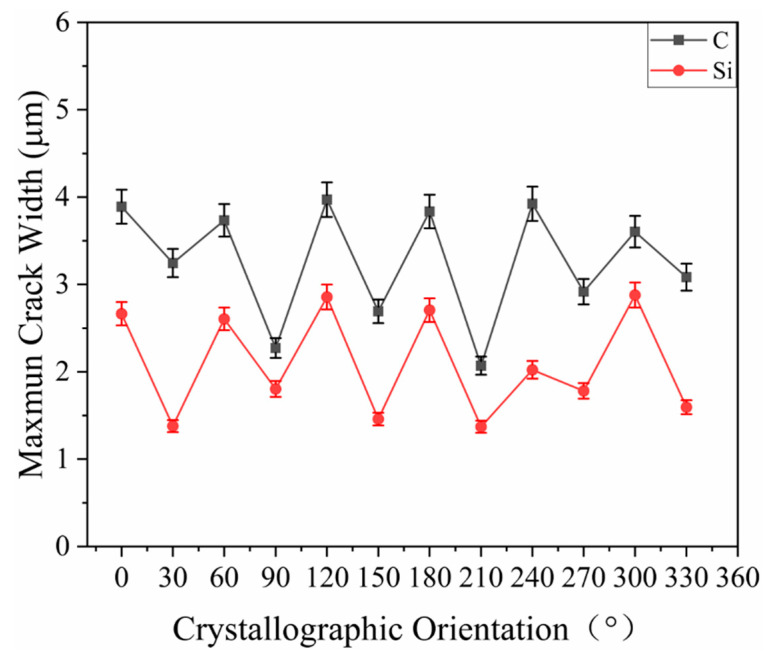
Maximum crack width of 4H-SiC.

Figure 11 shows the maximum width of cracks on the flanks of the scratch. As is shown in Figure 11, the curve of the C-plane was located above that of the Si-plane, and the <11$\bar{2}$0> direction of the C-plane was at the peaks of the curve with their values close to each other, while the <10$\bar{1}0$> direction was in the troughs of the curve. The fluctuation of the curve of the Si-plane was consistent with that of the C-plane, except for the 240° direction, where the values at the peak and in the troughs were relatively close for the C- and Si-planes;that is, the number and length of cracks flanking the stretch in the <11$\bar{2}$0> direction were greater than those in the <10$\bar{1}0$> direction. The average maximum width of cracks in the <11$\bar{2}$0> direction of the C-plane is 3.826 μm, which is larger than the average maximum width of cracks in the <10$\bar{1}0$> direction of 2.713 μm. The average maximum width of cracks in the <11$\bar{2}$0> direction of the Si-plane is 2.622 μm, which is greater than the average maximum width of cracks in the <10$\bar{1}0$> direction of 1.564 μm.

## 3. Discussion

From the above analysis, it can be seen that the elastic modulus is a measure of an object’s ability to resist elastic deformation. The larger the elastic modulus, the less likely the material is to deform and the harder and more rigid and brittle it is. The elastic modulus of the C-plane is greater than those of the Si-plane, so the Si-plane exhibits a greater elastic recovery at the same penetration depth, while the indentation residual depth and the maximum scratch residual depth of the C-plane are greater than the Si-plane. Because of this, the pop-in value of the C-plane is smaller than that of the Si-plane. Meanwhile, the C-plane has a smaller average length of the elastic deformation during scratching compared with the Si-plane, which also indicates a shorter elastic processing time. Therefore, more load is needed for the same penetration depth on the C-plane than on the Si-plane, which further requires larger lateral force on the C-plane under the same experimental conditions.

**Figure 12:**
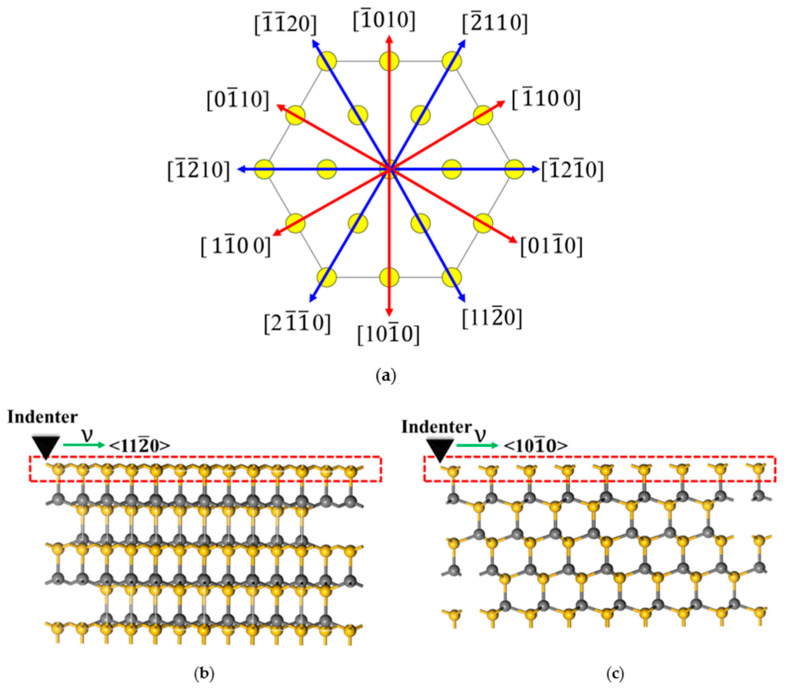
Lattice structure of 4H-SiC, (**a**)—arrangement of Si atoms on the Si-plane, (**b**)—scratch along the<11$\bar{2}$0> directions, (**c**)—scratch along the [$\bar{1}$100] directions.

The results from nanoscratch tests show that the curves of the maximum scratching force, the maximum residual depth of the scratch, and the maximum width of cracks for C- and Si-planes fluctuate with identical periods and exhibit a consistent trend for the two planes, both at the peak in the <11$\bar{2}$0> direction and in the trough in the <10$\bar{1}0$> direction. The values of the maximum residual depth of the scratch and the maximum width of the crack are close to each other either at the peak or in the trough. Figure 12 shows the lattice structure of 4H-SiC, Figure 12a the arrangement of Si atoms on the Si-plane of 4H-SiC, and Figure 12b,c Si atoms in the scratch along the [$\bar{1}$2$\bar{1}$0] and [$\bar{1}10$0] directions. As is shown in Figure 12a, it is due to the periodic nature of the lattice structure of 4H-SiC that atoms are arranged in the same way in the <11$\bar{2}$0> directions and in the <10$\bar{1}0$> directions as well. In addition, literature [16] shows that the distance between neighboring Si atoms is smaller in the [$\bar{1}$2$\bar{1}$0] direction than in the [$\bar{1}10$0] direction, which means that Si atoms are more tightly packed when scratching along the [$\bar{1}$2$\bar{1}$0] direction (Figure 12b,c), hence there is a larger maximum lateral force in the <11$\bar{2}$0> direction than in the <10$\bar{1}0$> direction. Moreover, atoms are arranged exactly in the same way in the C-plane as in the Si-plane, so the scratching force exhibits the same pattern. Since the C-C bond energy is larger than the Si-Si bond energy [12], however, a larger force is required when scratching is performed on the C-plane.

As to the material damage, at a certain cracking size, the larger the fracture toughness value of the material, the greater the critical stress required for its cracks to destabilize and expand, so cracks prefer the lowest energy route. It is found that the fracture toughness $k_{\mathrm{IC}}$ along the <11$\bar{2}$0> direction is 1.8 MPa.$m^{1∕2}$ and along the <10$\bar{1}0$> direction is 1.4 MPa.$m^{1∕2}$ [9]. When the scratch is made along a direction, the flanking stress intensity in a different direction first reaches its fracture toughness ($k_{\mathrm{IC}}$) value, causing cracking to the sides. Since the <11$\bar{2}$0> direction exhibits higher fracture toughness, it has more and longer cracks on both sides of the scratch. Therefore, compared with the <10$\bar{1}0$> direction, the <11$\bar{2}$0> direction has a greater maximum residual depth of the scratch and a greater number and length of cracks on both sides of the scratch.

## 4. Conclusions

In this paper, nanoindentation and scratch tests were carried out on different crystallographic planes and orientations of 4H-SiC. The differences in hardness, elastic modulus, and displacement-load curves of the C- and Si-planes, as well as in the morphologies and damage-related parameters of scratches in different crystallographic orientations of the C- and Si-planes were compared and analyzed. The causes of the phenomenon and the reasons for the divergence in scratches of different crystallographic planes and orientations were explained through the lattice structure. The main conclusions are as follows.

According to the test results of this paper, it can be known that the C-plane of 4H-SiC is more rigid, while the Si-plane is more elastic and ductile. As the C-plane undergoes a smaller elastic-plastic processing phase than that of the Si-plane, better surface quality may be obtained in the nanoscale abrasive machining of the Si-plane, but the material removal is relatively more difficult.

The removal of 4H-SiC material during the brittle-ductile transition phase is mainly characterized by the appearance of tearing along the bottom of the scratch grooves, followed by micro-cracks on both sides of the grooves, which tend to expand in the direction of low fracture toughness, and eventually by the flanking cracks to expand, deflect, and connect, causing spalling of the material. 

The maximum lateral forces, maximum residual depths of scratches, and maximum widths of cracks in different crystallographic orientations on the C- and Si-planes of 4H-SiC are obviously periodic in the crystallographic orientations at 30° intervals.

Scratching along the <11$\bar{2}$0> direction on both the C- and Si-planes of 4H-SiC is more prone to crack expansion. Relatively speaking, better machined surface quality is easily obtained along the <10$\bar{1}0$> direction on the C- and Si-planes.

## Figures and Tables

**Table 1:** Nano Indenter G200 key specifications.

Main Technical Indicators	Parameter
XP mode displacement resolution	<0.01 nm
XP mode load resolution	<50 Nn
DCM mode displacement resolution	<0.0002 nm
DCM mode load resolution	<30 Nn
Scratch speed	0.1 µm/s to 2.5 mm/s
Maximum lateral force resolution	2 µN

**Table 2:** Parameters of nanoscratch tests.

Test Parameters	Numerical Value
Fillet radius of indenter	5 μm
Maximum load	200 Mn
Scratch speed	10 μm/s
Length of scratches	50 μm

## Data Availability

The data that support the findings of this study are available from the corresponding author, N.D. (Nian Duan), upon reasonable request.
